# A literature-based approach for curating gene signatures in multifaceted diseases

**DOI:** 10.1186/s12967-020-02408-7

**Published:** 2020-07-10

**Authors:** Mathieu Garand, Manoj Kumar, Susie Shih Yin Huang, Souhaila Al Khodor

**Affiliations:** Research Department, Sidra Medicine, Doha, Qatar

## Abstract

**Background and aims:**

The task of identifying a representative and yet manageable target gene list for assessing the pathogenesis of complicated and multifaceted diseases is challenging. Using Inflammatory Bowel Disease (IBD) as an example, we conceived a bioinformatic approach to identify novel genes associated with the various disease subtypes, in combination with known clinical control genes.

**Methods:**

From the available literature, we used Acumenta Literature Lab^TM^ (LitLab), network analyses, and LitLab Gene Retriever to assemble a gene pool that has a high likelihood of representing immunity-related subtype-specific signatures of IBD.

**Results:**

We generated six relevant gene lists and 21 intersections that contain genes with unique literature associations to Crohn’s Disease (n = 60), Ulcerative Colitis (n = 17), and unclassified (n = 45) subtypes of IBD. From this gene pool, we then filtered and constructed, using network analysis, a final list of 142 genes that are the most representative of the disease and its subtypes.

**Conclusions:**

In this paper, we present the bioinformatic construction of a gene panel that putatively contains subtype signatures of IBD, a multifactorial disease. These gene signatures will be tested as biomarkers to classify patients with IBD, which has been a clinically challenging task. Such approach to diagnose and monitor complicated disease pathogenesis is a stepping-stone towards personalized care.

## Background

Inflammatory Bowel Disease (IBD) is an inflammatory disorder of the gastrointestinal tract (GIT), resulting from the complex interactions between host (genetic, immune responses) and environment (external factors, microbiota) [1]. IBD is characterized by repeated alternating cycles of clinical relapse and remission, and in the absence of adequate treatments, a chronic inflammation leading to irreversible intestinal damages [2]. IBD is classified into three major subtypes [3]: Ulcerative Colitis (UC), which primarily affects the colon, Crohn’s Disease (CD), which affects various GIT sites [4], and a third subtype where histology assessments do not categorize to either UC or CD. The latter subtype is defined as “Inflammatory Bowel Disease, type unclassified” (IBDU) [5, 6].

**Fig. 1:**
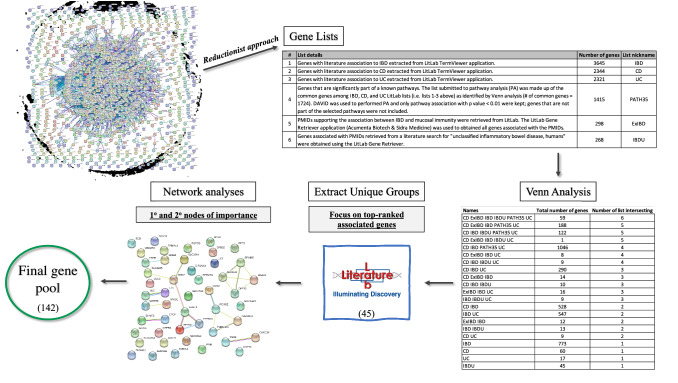
Workflow of constructing a gene panel that putatively contains subtype signatures of Inflammatory Bowel Disease (IBD). **a** network of the predicted protein–protein interactions (STRING) inferred from the genes associated with immune responses and IBD highlights the complexity and difficulty of making logical interpretation (network image in top-left corner). In order to simplify the process, we devised six gene lists from different sources. Details on the methods used to retrieve the genes, the number of genes in the lists, and the terms used onwards are shown (Gene Lists). Then, we submitted the lists to Venn analysis which resulted in 21 intersections (Venn Analysis). The common (i.e. IBD core genes) and the unique lists for CD, UC, and IBDU were submitted to Literature Lab to obtain pathways and diseases association scores; each gene is ranked based on its contribution weight to a score (Extract Unique Groups–Literature Lab). In the network analysis, the top-ranking genes (those with > 5% contribution to the association) informed which nodes to expand to the primary and secondary nodes. We used both GeneMania and STRING (shown here) to obtain those networks. In the network shown, the edges depict the known protein interactions based on knowledge from curated databases (blue edges), experimentally determined (pink edges), and co-expression data (black edges). Genes without shared pathways are shown as independent nodes. The amalgamation of the gene selections from all the common and subtypes-specific genes amounts to 142 putative target genes

A rapid increase in global incidences of UC and CD was observed after World War II, particularly in industrialized countries (www.crohnscolitisfoundation.org). Currently, IBD affects around 5 million people worldwide and is expected to increase steadily over the next decade [7]. Classifying IBD patients has been challenging due to disease heterogeneity and its various atypical phenotypes [8]. Although the mechanisms underlying IBD pathogenesis are not fully understood, an overactive mucosal immune response and a dysbiotic gut microbiome are commonly observed among IBD patients [9, 10]. Endoscopy and colonoscopy are the current methods used for differentiating CD and UC but they carry the risks of bowel perforation and infections. Non-invasive routine laboratory investigations, on the other hand, cannot independently and reliably ascertain diagnosis [11]. These conventional diagnoses have high incidences of IBD subtype misdiagnosis and often lead to unsatisfactory patient outcomes and unnecessary treatments. Although genes have been identified to be involved in IBD pathogenesis, the sheer volume of the associations poses a difficulty for defining a molecular signature of the disease and its subtypes. As an example, around 40 studies relating to IBD gene biomarkers have been indexed on PubMed on a yearly basis over the last 10 years. Furthermore, the known signaling pathways involved in the immune responses in IBD patients are extremely complex (Fig. 1). Despite these challenges, it is critical to understand the molecular signature specific to each IBD subtype in order to provide the most appropriate and personalized care for IBD patients.

We hypothesized that IBD-subtype signatures can be identified by a small but representative set of genes. In a reductionist approach, we performed a stepwise method to construct a representative molecular signature driven by the contribution of individual genes to the current literature. Our overall strategy reduces the complexity and number of potential gene targets by intersecting multiple relevant gene lists. This novel approach allows the reiterative process of filtering and focusing on unique (potential subtype signature) as well as common IBD genes (i.e. core genes involved in IBD pathogenesis and serving as positive controls). The workflow of our method is as shown in Fig. 1. In short, we applied an intelligent and informed selection strategy to design a “targeted” transcriptomic assay for the diagnosis of IBD subtypes and monitoring of its pathogenesis.

## Results and discussion

Despite the vast amount of transcriptomic data generated in the past decade, specific IBD subtype signatures have not been clearly identified. However, the available data is a valuable resource for large-scale mining of genes associated/reported with IBD. Systems biology and reductionist approaches have identified several key genes or pathways for determining and characterizing the cause and progression of IBD [12, 13]. However, extrapolating and summarizing the results have been difficult due to the heterogeneity of datasets and experimental designs. Yet, having a robust and encompassing gene signature will add enormous practical value in today’s clinic. With the need to reduce the complexity of an ever-growing pool of potential biomarkers, we present here the dissection of the putatively unique and common gene lists for IBD, queried by a combined approach using a wide-scale literature mining, network analysis, and gene ontology tools.

To answer the question: “what is known about the genes associated with each IBD subtypes?”, we employed a novel literature mining approach to query and agglomerate multiple relevant gene lists sourced from the literature, and from which unique immune signature associated with CD, UC, IBDU, or IBD in general can be extracted. In brief, we conducted a statistical association analysis of genes with literature (PubMed) using the Term Viewer (for IBD, CD, UC) and Gene Retriever (for IBDU) functions of LitLab [14]. A list of genes common among IBD, CD, and UC (i.e. Lists 1–3, Fig. 1—Gene lists) was submitted to pathway analysis (PA) in order to capture additional interacting genes (neighboring nodes). DAVID (137, 138) was then used to performed PA, and only genes with an association *p*-value < 0.01 were kept (List 4, Fig. 1—Gene lists). LitLab Gene Retriever application (https://www.acumenta.com/generetriever) retrieves genes associated with a publication list and is useful in specific search strategies or under less known conditions, as for the case of IBDU. As IBD etiology has a major immune component [12], we focused our targeted panel by retrieving genes associated with PubMed IDs (PMIDs) supporting the association between IBD and mucosal immunity (List 5, Fig. 1—Gene lists). In addition, we retrieved all the genes associated with PMIDs pertaining to IBDU in humans (List 6, Fig. 1—Gene lists), where the LitLab Gene Retriever outputs are provided in Additional file 1: Table S1.

**Fig. 2:**
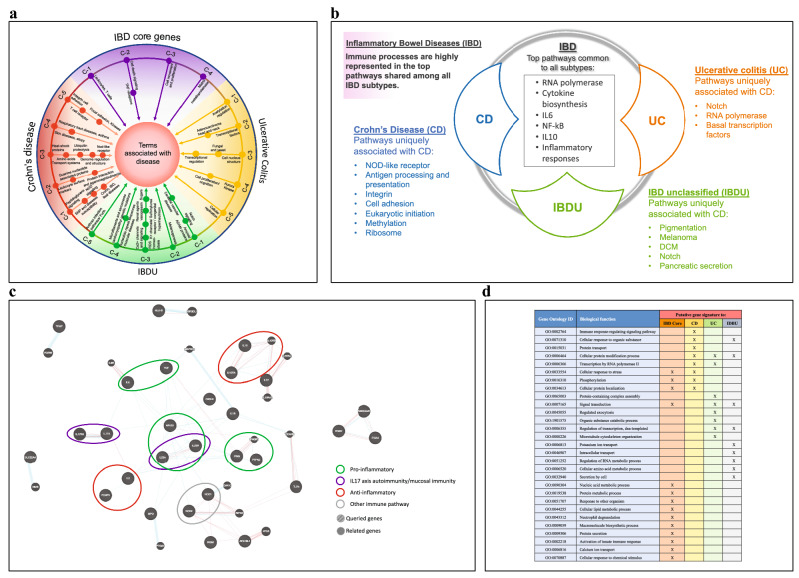
Literature-based assessment of Medical Subject Headings (MeSH) terms, pathways, and Gene Ontology (GO) annotation associated with IBD subtype-specific gene lists. **a** Representation of MeSH terms associated with each IBD disease subtypes identified using our data mining approach. Briefly, genes representing IBD in general, and those uniquely related to each subtype, were identified in our Venn analysis. Each list of genes was submitted to cluster analysis using Literature Lab PLUS; clusters (outside ring labelled C-1, 2, 3, etc.) and sub-clusters (large dot on lines connected to a cluster) of MeSH terms for each disease group are shown. IBD, Inflammatory Bowel Diseases; *CD* Crohn’s Diseases: *UC* Ulcerative colitis: *IBD-U* IBD unclassified. **b** Visualization of the associated pathways in each disease subtype and with the IBD core genes. Among the pathways common to IBD and all subtypes, immune processes are highly represented. **c** Network focused on the key factors and major pathways involved in IBD(s) pathogenesis. The top 50 common associated genes between IBD-CD, and UC were used to generate a network map. Blue connecting lines represent pathways (i.e. the 2 genes connected share a pathway) and red lines represent physical interaction. Genes without connections are not shown in the image. Colored ellipses highlight key immune pathways: pro-inflammatory (green), IL17 (purple), and anti-inflammatory (red). **d** Gene set annotations with GO. GO terms significantly associated with each disease subtype were identified using GSAn and are indicated by a “X”

To evaluate the unique and overlapping genes, the six gene lists were subjected to Venn analysis which resulted in 21 intersections (Fig. 1—Venn analysis**)**. The unique gene lists identified for each disease subtype (i.e. genes with literature associations to only one of the subtypes; gene lists provided in Additional file 2: Figure S1) were then used as input in LitLab Gene Editor/PLUS applications in order to determine the significant MeSH term associations (summarized in Fig. 2a). Genes contributing to more than 5% to a term association were highlighted as important and carried forward for further exploration in subsequent network analysis; 45 genes were selected at this stage (Fig. 1—Literature lab**)**. By focusing on gene associations in Pathways and Diseases domains, we filtered for significant signaling mechanisms specific for an IBD disease subtype and, as well, shed light on possible novel interactions.

Our analysis revealed that pathways involved in T cell receptor signaling, integrin signaling, NOD-Like Receptors (NLR) signaling, ubiquitin-mediated proteolysis, and cell adhesion were specifically associated with CD (summarized in Fig. 2b). Some of these pathways have experimental validations, which added strength to our search strategy and confirmed our findings. For example, integrin is considered a potential therapeutic target for CD in clinical trials [15]. It is also known that the most predominant link to the onset of CD is a genetic mutation in the innate immune receptor (NOD2) [16]. While for the other pathways that we have identified, their contributions to IBD pathogenesis warrants future research. Overall, the network of the top common genes associated with IBD (Fig. 2c) shows immune-concentrated relations around the NOD, IL23, IL17, IFNG, IL6/TNF, TLR4/IL1B, and IL10 pathways.

Network analyses of the disease subtype-specific genes were used to expand our gene panel to include neighbor genes by mean of physical interaction or biological pathways (Fig. 1). Network formation allows the identification of relationship between genes that are known to be associated/contributing to the pathways of interest. Such relationships are needed to identify important biological and putative molecular events driving each IBD subtype. As such, we were able to expand the final panel to include genes corresponding to the primary and secondary nodes of our initial gene list. For example, in CD, the NOD2 genes, which majorly contributed to the specific Pathways and Diseases associations, were found to be associated with genes such as SUGT1, ECD, and EIF2AK4 that are involved in cell cycle progression and protein translation. This observation indicates a putative link between the innate immune receptor and the control of cellular growth. Those genes have been shown to promote cell proliferation and migration of human airway smooth muscle cells [17] and are expressed in high levels in proliferating colonic epithelial cells [18]. In addition, these genes may help to facilitate key functions of NOD2 in intestinal epithelial and hematopoietic cells [19]. At the end, the method described above produced a final list composed of 142 genes.

Additional gene set annotations with Gene Ontology (GO) was performed using GSAn and revealed subtle differences in the important biological process enriched among each IBD subtype (Fig. 2d). Although GO enrichment alone does not provide the granularity required to differentiate the disease subtype, the biological enrichment of genes most important for each subtype do allow the detection of some molecular differences. For example, immune response signaling and phosphorylation events distinguished CD annotation from other subtypes and recapitulated the prominent involvement of the NOD2 pathway in the pathogenesis of CD. In combination with the other tools that we have implemented, GO provided an additional support to the relevance of our selected gene panels.

We executed a thorough mining of the literature for gene association with IBD subtypes, which resulted in the construction of a gene panel that putatively contains IBD subtype signatures. In order to provide support that our results hold promises for discovery of IBD subtype biomarker/signature, we performed preliminary analyses of published datasets comparing CD and UC cohorts. In the first dataset, GSE3365, patients with Crohn’s disease (CD = 59) and ulcerative colitis (UC = 26) were compared. Healthy controls segregated clearly from either CD or UC, however clustering of CD and UC by PLSDA did not pass our validation threshold of Q2 > 0.4. We then performed ANOVA and selected specifically the statistically significant genes followed by hierarchical clustering, from which we observed separation based on group average (Additional file 2: Figure S2). In GSE6731, colonoscopic biopsies from patients with Crohn’s Disease (CD = 19) or lUcerative Colitis (UC = 8) were compared. Hierarchical clustering, based on the average per group, showed noticeable segregation and subset-specific clustering based on the expression of our gene panel (data not shown). The PLSDA results showed a modest but valid segregation of samples: permutation (2000), p-value < 0.003, Q2 > 0.4 (Additional file 2: Figure S2). Together, these preliminary results provided indications that CD and UC patients would segregate based on the expression profile of genes, or a subset of, contained in the gene panel. We believe the classification would considerably improve with the addition of metadata (i.e. clinical assessment of severity and/or disease phase) into a statistical modeling framework.

## Conclusions

In this article, we proposed the application of a novel gene pool enrichment methodology for mining IBD subtype signatures. Such refined disease signatures could provide novel and unbiased diagnostic avenues and help to identify subtype-specific biomarkers that can be non-invasive, highly specific, reliable and easy to assess by clinicians in routine practices. A similar approach can be applied to other multifaceted diseases and those with challenging triage in the clinic. While the process greatly reduced the scale of the search, validation of  the proposed genes in specific population cohort is still warranted. The latter effort is currently underway with our IBD cohort study. In this cohort, targeted transcriptomic including the genes mentioned in this article, along with other omics, will be employed to derive biomarker signatures. Preliminary results from this cohort showed that the gene panel contained the necessary element to differentiate the transcriptomic signatures of UC patients in remission versus flare (data not shown). Nevertheless, our results may provide a promising base for future transcriptomic analyses, in the context of extrapolating and summarizing publicly available gene biomarker studies or multi-omics approach-type of studies [12].

## Methodology

### Acumenta Literature Lab^TM^ (LitLab)

LitLab (Acumenta Biotech, NY, USA; http://www.acumenta.com/) [14] allows the identification of biological and biochemical terms that are significantly associated in the literature with a gene set, providing meaning to experimentally derived genes and proteins of interest. Unlike other enrichment tools, LitLab does not depend on fixed databases or a priori determinations. LitLab distinguishes itself by calculating the product log of the frequency of the input genes with the terms against the 86,000 terms in the Literature Lab™ database, which contains all the genes, biological and biochemical terms referenced in every PubMed abstract (as of 20 Jan 2020). The results are compared with those of 1000 random gene sets to compute statistical significance. Pathway and Diseases MeSH Terms associations were obtained using LitLab and contained scores. All reported associations corresponded to “Strong” or “Moderate”, which are defined as a score > 2.0 which is equivalent to p-value < 0.0228 or a score > 1.5 which is equivalent to p-value < 0.0668, respectively. Other qualifier values are shown in Additional file 2: Figure S3a and detailed definitions can be obtained from LitLab's extensive help files. This score represents the number of standard deviations away from the mean score obtained with the 1000 random gene lists . We created a table that summarized the scores, along with other metrics calculated by LitLab, obtained for each pathway associated with IBD subtypes (see Additional file 2: Figure S3b).

LitLab is composed of four main applications: Term Viewer, PLUS, Editor, and Gene Retriever. The LitLab Term Viewer application was used to extract gene associated with literatures published from 01/01/1990 until 30/09/2019 for the following terms: Inflammatory Bowel Disease, Ulcerative Colitis, and Crohn’s Disease. The returned articles were also reviewed for additional relevant articles. LitLab Gene Editor and PLUS applications were then used on the gene lists to obtain the association scores, which rank the genes based on their contribution weights to the score. Focus was placed on Pathways and Diseases as the domains are the most useful/relevant for extrapolating the role of the selected genes in the context of IBD. LitLab Gene Retriever is a data mining solution to retrieve all genes associated with a list of PubMed articles. Gene Retriever processes a list of PubMed IDs and produces an analysis of the genes mentioned in the title, text, and MeSH tags of each article. Results are then statistically ranked and presented in a spreadsheet to enable quick and comprehensive analyses.

The results and methods assisted by LitLab are all available to the users, making it transparent and traceable. The precision and recall (i.e. sensitivity) of any query are driven by the content used in the search. LitLab searches are assisted by a built-in gene thesaurus which creates an exhaustive queries of the literature. LitLab Gene Thesaurus regularly mines NCBI, UniProt, HUGO, and other annotation repositories to gather the broadest set of terms (aliases) for genes. Therefore, literature Lab searches are based on automated interrogation of term co-occurrence, leveraging the tools and tagging built into PubMed by NCBI, along with the powerful Literature Lab Gene Thesaurus and its formation of searches beyond the skills and time availability of most scientists.

### PubMed-based literature searches

PubMed search for IBDU utilized the following terms/strategies: “unclassified inflammatory bowel disease”[All Fields] OR (Intermediate[All Fields] AND (“inflammatory bowel diseases”[MeSH Terms] OR (“inflammatory”[All Fields] AND “bowel”[All Fields] AND “diseases”[All Fields]) OR “inflammatory bowel diseases”[All Fields] OR (“inflammatory”[All Fields] AND “bowel”[All Fields] AND “disease”[All Fields]) OR “inflammatory bowel disease”[All Fields])) AND “humans”[MeSH Terms]. PubMed search for “immune response and inflammatory bowel diseases” utilized the following terms/strategies: (“inflammatory bowel diseases”[All Fields] OR “IBD”[All Fields]) AND (“immune responses”[All Fields] OR “immune”[All Fields] OR “immunity”[All Fields]) AND “humans”[MeSH Terms].

### Network analyses

Network analysis of the relationships among our significant weighted genes based on biological processes was performed using GeneMania [20]; the edges denote both physical interactions (orange) and pathways (blue). Network of the predicted protein–protein interactions inferred from our significant gene list was performed using STRING [21]. The edges depict the known protein interactions based on knowledge from various curated databases (blue edges), experimentally determined (pink edges), and co-expression data (black edges).

### Gene ontology

Gene ontology (GO) enrichment analysis was performed on the specific gene signatures identified from our data mining approach. Gene set annotations with  GO terms was performed using GSAn (refer to 10.1109/iv.2017.18 and https://gsan.labri.fr/start for details) [22]. Briefly, GSAn combines data mining and combinatorial algorithms to provide a reduced and synthetic number of GO terms describing the biological roles of the input gene group as a whole.

### Statistical analysis

ANOVA, hierarchical clustering, and heatmap visualization were performed in MetaboAnalyst (4.0).

## Supplementary information

**Additional file 1: Table S1**. LitLab Gene Retriever output of genes retrieved from PMIDs associated with IBD and mucosal immunity, and IDBU in humans.

**Additional file 2: Figure S1**. List of genes with unique literature associations to IBD subtypes. Gene lists were generated as shown in Fig. 1 and described in Methods. **Figure S2**. Preliminary analyses of published datasets comparing CD and UC cohorts; GSE3365 and GSE6731. **Figure S3**. **a** Description of Pathways MeSH Terms association scores and other qualifier values in LitLab. **b** Summary table of the scores, along with other metrics calculated by LitLab, for each literature-based Pathways association for the IBD subtypes.

## Data Availability

The datasets used and/or analyzed during the current study are available from the corresponding author on reasonable request.
