# Supplementary material for: A literature-based approach for curating gene signatures in multifaceted diseases
Source: J Transl Med. 2020 Jul 10;18:279. doi: 10.1186/s12967-020-02408-7 (PMC7350750; doi:10.1186/s12967-020-02408-7)
Supplement: Supplementary file 2 — Additional file 2: Figure S1. List of genes with unique literature associations to IBD subtypes. Gene lists were generated as shown in Fig. 1 and described in Methods. Figure S2. Preliminary analyses of published datasets comparing CD and UC cohorts; GSE3365 and GSE6731. Figure S3. a Description of Pathways MeSH Terms association scores and other qualifier values in LitLab. b Summary table of the scores, along with other metrics calculated by LitLab, for each literature-based Pathways association for the IBD subtypes. [file 12967_2020_2408_MOESM2_ESM.docx]

**A literature-based approach for curating gene signatures in multifaceted diseases**

Mathieu Garand^1^*, Manoj Kumar^1^, Susie SY Huang^1^, Souhaila Al Khodor^1^*

^1^Research Department, Sidra Medicine, Doha, Qatar

* Address correspondence to Dr. Mathieu Garand: mathieu.garand@gmail.com and Dr. Souhaila Al Khodor: salkhodor@sidra.org

**Additional file 2**

**Additional file 2: figure S1**. List of genes with unique literature associations to IBD subtypes. Gene lists were generated as shown in Figure 1 and described in Methods. Briefly, genes associated with CD and UC were obtained via Literature Lab Gene Thesaurus-leveraged PubMed queries and IBDU via retrieval of genes from PMID using Literature Lab Gene Retriever. Altogether with lists from 3 other sources, genes were submitted to Venn analysis to identify those associated with each IBD subtypes.

**GSE3365** **GSE6731**

**Additional file 2**: Figure S2 Preliminary analyses of published datasets comparing CD and UC cohorts; GSE3365 and GSE6731. A subset of the genes within our panel that were significantly differentially expressed between CD and UC(ANOVA <0.01) were used in hierarchical clustering analysis. Separation based on the average per group was clearly observed. In GSE6731, colonoscopic biopsies from patients with Crohn’s disease (CD=19) or ulcerative colitis (UC=8) were compared. Our PLSDA results showed a modest but valid segregation of samples: permutation (2000), p-value<0.003, Q2>0.4

**A)**

**B)**

**Additional file 2: Figure S3**. A) Description of Pathways MeSH Terms association scores and other qualifier values in LitLab. B) Summary table of the scores, along with other metrics calculated by LitLab, for each literature-based Pathways association for the IBD subtypes.
